# Is the optimal examined lymph node number for rectal cancer altered?

**DOI:** 10.1097/JS9.0000000000001427

**Published:** 2024-04-09

**Authors:** Xiaoyan Li, Ke Hu

**Affiliations:** Department of Radiation Oncology, Peking Union Medical College Hospital, Chinese Academy of Medical Sciences & Peking Union Medical College, Beijing, People’s Republic of China

*Dear Editor,*

Surgery is the primary treatment for rectal cancer (RC), and it remains the cornerstone of curative intent. The examination of lymph nodes is an essential aspect of radical surgery for RC and directly reflects the quality of the procedure. Many studies have demonstrated a significant association between the count of examined lymph nodes (ELNs) and the prognosis of RC. To ensure an adequate resection, the American Joint Committee for Cancer guidelines recommend the examination of a minimum of 12 lymph nodes in colorectal cancer specimens. However, Guan *et al*.^1^ analyzed the correlation between ELN count and overall survival (OS) in RC by using the Surveillance, Epidemiology, and End Results (SEER) database and a Chinese multi-institutional registry. They discovered that a higher ELN count is related to more accurate nodal staging and improved survival in RC. The optimal cut-off point for assessing the quality of lymph node examination and prognosis stratification is 15 ELNs. While the authors have acknowledged the limitations in the ‘Discussion’ section, we have provided insightful recommendations for additional improvement.

Firstly, a variety of surgical approaches, depending on the location and extent of the disease, are used to treat primary RC lesions. In cases of T1N0 RC, local resection can be a viable treatment option following careful screening. However, for the majority of RCs, a standardized approach to radical surgery is recommended. Total mesorectal excision (TME) has been shown to yield favorable outcomes and is therefore considered a best practice^2^. Ample evidence exists to support that the quality of RC resection is crucial and affects local recurrence and survival. The prognosis of patients undergoing surgical treatment is influenced by the performance of standard radical surgery. So if this study is to establish a quantitative correlation between the count of ELN and the long-term survival in RC, it is imperative to critically assess whether the patients underwent standard radical surgery. However, the study did not include an assessment of the quality of surgery in the patients. This may lead to a distortion of the true correlation between the number of ELN and survival.

Secondly, to bolster the reliability of the findings, the researchers made efforts to account for potential variables that could impact the results. Nonetheless, given the constraints of the SEER database, there remain some critical prognostic factors that were not taken into account, such as resection margin, tumor location, and extramural vascular invasion (EMVI)^3^. The study’s findings are somewhat hindered by these limitations, which may affect their credibility and applicability. It is important to take into account these constraints when interpreting the results and drawing conclusions.

In conclusion, we acknowledge and appreciate the authors’ diligent efforts to conduct a large-scale retrospective study on RC. However, we are of the opinion that further research is necessary to ascertain the optimal number of ELN for long-term survival.

## Ethical approval

This article does not require any human or animal subjects to acquire such approval.

## Consent

This article does not require any human or animal subjects to acquire such approval.

## Sources of funding

This study is supported by National Key R&D Program of China, Ministry of Science and Technology of the People's Republic of China (No. 2022YFC2402305).

## Author contribution

X.L.: conceptualized, reviewed, and edited; X.L. and K.H.: wrote, made the draft, and updated it. All authors have critically reviewed and approved the final draft and are responsible for the content and similarity index of the manuscript.

## Conflicts of interest disclosure

The authors declare no conflicts of interest.

## Research registration unique identifying number (UIN)

Not applicable.

## Guarantor

All authors.

## Data availability statement

No data were generated during the writing of this study.

## Provenance and peer review

Not commissioned, externally peer-reviewed.
